# Does interaction matter? Testing whether a confidence heuristic can replace interaction in collective decision-making

**DOI:** 10.1016/j.concog.2014.02.002

**Published:** 2014-05

**Authors:** Dan Bang, Riccardo Fusaroli, Kristian Tylén, Karsten Olsen, Peter E. Latham, Jennifer Y.F. Lau, Andreas Roepstorff, Geraint Rees, Chris D. Frith, Bahador Bahrami

**Affiliations:** a Interacting Minds Centre, Aarhus University, Jens Chr. Skous Vej 4, Building 1483, 8000 Aarhus, Denmark; b Department of Experimental Psychology, University of Oxford, South Parks Road, Oxford OX1 3UD, United Kingdom; c Calleva Research Centre for Evolution and Human Sciences, Magdalen College, High Street, Oxford OX1 4AU, United Kingdom; d Center for Semiotics, Aarhus University, Jens Chr. Skous Vej 2, Building 1484, 8000 Aarhus, Denmark; e Gatsby Computational Neuroscience Unit, UCL, 17 Queen Square, London WC1N 3AR, United Kingdom; f Institute of Cognitive Neuroscience, UCL, 17 Queen Square, London WC1N 3AR, United Kingdom; g Wellcome Trust Centre for Neuroimaging, UCL, 12 Queen Square, London WC1N 3BG, United Kingdom; h All Souls College, University of Oxford, High Street, Oxford OX1 4AL, United Kingdom

**Keywords:** Collective decision-making, Interaction, Confidence, Reaction time, Heuristic, Perception, Metacognition, Signal detection theory, Computational

## Abstract

In a range of contexts, individuals arrive at collective decisions by sharing confidence in their judgements. This tendency to evaluate the reliability of information by the confidence with which it is expressed has been termed the ‘confidence heuristic’. We tested two ways of implementing the confidence heuristic in the context of a collective perceptual decision-making task: either directly, by opting for the judgement made with higher confidence, or indirectly, by opting for the faster judgement, exploiting an inverse correlation between confidence and reaction time. We found that the success of these heuristics depends on how similar individuals are in terms of the reliability of their judgements and, more importantly, that for dissimilar individuals such heuristics are dramatically inferior to interaction. Interaction allows individuals to alleviate, but not fully resolve, differences in the reliability of their judgements. We discuss the implications of these findings for models of confidence and collective decision-making.

## Highlights

• We tested whether a confidence heuristic could replace interaction in a collective perceptual decision-making task.
• For individuals of nearly equal reliability, the confidence heuristic is just as accurate as interaction.
• For individuals with different reliabilities, the confidence heuristic is less accurate than interaction.
• Interacting individuals use the credibility of each other’s confidence estimates to guide their joint decisions.
• Interacting individuals face a problem of how to map ‘internal’ variables onto ‘external’ (shareable) variables.

## 1 Introduction

There is a growing interest in the mechanisms underlying the “two-heads-better-than-one” (2HBT1) effect, which refers to the ability of dyads to make more accurate decisions than either of their members (e.g., Hill, 1982). One study (Bahrami et al., 2010), using a perceptual task in which two observers had to detect a visual target, showed that two heads become better than one by sharing their ‘confidence’ (i.e., an internal estimate of the probability of being correct), thus allowing them to identify who is more likely to be correct in a given situation. Sharing of confidence as a strategy for combining individual opinions into a group decision has also been established in non-perceptual domains (e.g., Sniezek & Henry, 1989). This tendency to evaluate the reliability of information by the confidence with which it is expressed has been termed the ‘confidence heuristic’ (e.g., Thomas & McFadyen, 1995).

A recent study has shown that a simple algorithm based on the confidence heuristic – always opt for the opinion made with higher confidence – can yield a 2HBT1 effect in the absence of any interaction between individuals (Koriat, 2012). Intrigued by this finding, we tested whether this algorithm could in practice replace interaction in collective decision-making. Importantly, such a formula for collective choice – if effective – would not be susceptible to the egocentric biases that may impair interaction (e.g., Gilovich, Savitsky, & Medvec, 1998), and could readily be used by decision makers, such as jurors, medical doctors or financial investors, who have to combine different opinions in limited time. Indeed, the implementation of heuristics inspired by individual decision-making has proved very useful within professional contexts (e.g., Gigerenzer, 2008).

### 1.1 Circumventing interaction

Building on Bahrami et al.’s (2010) study, Koriat (2012) asked isolated observers to estimate the degree of confidence in their perceptual decisions. Participants, all of whom had received the same sequence of stimuli, were afterwards paired into virtual dyads so that they matched each other in terms of their ‘reliability’ (i.e., the reliability of their individual decisions about the visual target). To remove individual biases in confidence, their confidence estimates were normalised, so that they shared the same mean and standard deviation, before being submitted to the Maximum Confidence Slating (MCS) algorithm, which selected the decision of the more confident member of the virtual dyad on every trial. While circumventing interaction, the MCS algorithm yielded a robust 2HBT1 effect. Interestingly, isolated observers’ confidence estimates are negatively correlated with their reaction times when responses are given in the absence of speed pressure (e.g., Patel, Fleming, & Kilner, 2012; Pleskac & Busemeyer, 2010; Vickers & Packer, 1982), raising the possibility that a Minimum Reaction Time Slating (MRTS) algorithm may be sufficient to yield a 2HBT1 effect.

In this study, we tested the efficacy of the MCS and MRTS algorithms without matching dyad members in terms of their reliability, and compared the responses advised by the algorithms with those reached by the dyad members through interaction (henceforth ‘dummy’ versus ‘empirical’ dyads/decisions). In particular, we addressed three questions. First, does the success of the MCS and MRTS algorithms depend on the similarity of dyad members’ reliabilities? Bahrami et al. (2010) found that the success of interactively sharing confidence was a linear function of the similarity of dyad members’ reliabilities. For similar dyad members, two heads were better than one. However, for dissimilar dyad members, two heads were worse than the better one. Interestingly, Bahrami et al. (2010) found that these discrepant patterns of collective performance could be explained by a computational model in which confidence was defined as a function of the reliability of the underlying perceptual decision. We predicted that the efficacy of the MCS and the MRTS algorithms would also depend on the similarity of dyad members’ reliabilities.

Second, do the algorithms fare just as well as interacting dyad members? People vary in their ability to estimate the reliability of their own decisions (e.g., Fleming, Weil, Nagy, Dolan, & Rees, 2010; Song et al., 2011); this ability is typically referred to as ‘metacognitive’ ability and, in social contexts, determines the credibility of people’s confidence estimates. While the algorithms are prone to error when people misestimate the reliability of their own decisions, interacting individuals may take such misestimates into account (e.g., Tenney, MacCoun, Spellman, & Hastie, 2007). We predicted that interacting dyad members would take into account the credibility of each other’s confidence estimates when making their joint decisions, and that interaction would be relatively more beneficial than the algorithms for dissimilar dyad members; they have more to lose from following the more confident but less competent of the two.

Third, what is the effect of normalising confidence estimates before selecting the decision made with higher confidence? Koriat (2012) reported that the MCS algorithm performed equally well when using raw and normalised confidence estimates as its input. However, this analysis was limited to (virtual) dyad members of nearly equal reliability. Even though people vary in the ability to evaluate the reliability of their own decisions, confidence estimates are rarely uninformative about underlying performance (e.g., Lau & Maniscalco, 2010). As a consequence, normalising confidence estimates may remove statistical moments that reflect actual differences in underlying performance (e.g. differences in average confidence due to differences in average performance). We therefore predicted that submitting normalised confidence estimates to the MCS algorithm would be relatively more costly for dissimilar dyad members.

## 2 Methods

### 2.1 Data and participants

To test our predictions, we analysed data from an experiment (Bahrami et al., 2012a) in which dyad members estimated their confidence in individual decisions on every trial, but were also required to make a joint decision whenever their individual decisions conflicted. We used data from two experimental conditions: a ‘non-verbal’ condition in which dyad members made their joint decisions only having access to each other’s confidence estimates, and a ‘verbal’ condition in which dyad members also had the opportunity to verbally negotiate their joint decisions (the NV condition and the NV&V condition in Bahrami et al., 2012a, 2012b). In total, fifty-eight participants (29 dyads) took part in the non-verbal (14 dyads) and the verbal (15 dyads) conditions. All participants were healthy adult males (*mean* age = 23.5 years, *SD* = 2.8 years) with normal or corrected-to-normal vision. The members of each dyad knew each other before taking part in the experiment. We describe the key experimental details below (see Bahrami et al., 2012a, 2012b, for response, display and stimulus parameters).

### 2.2 Experimental details

Dyad members sat at right angles to each other in a dark room, each with their own screen and response device. For each trial, dyad members viewed two brief intervals, on which six contrast gratings were presented simultaneously around a central fixation point. In either the first or the second interval, one of the six contrast gratings had a slightly higher level of contrast.

After the two viewing intervals, a horizontal line with a fixed midpoint appeared on each dyad member’s screen. The left side of the midpoint represented the first interval, the right side represented the second. An additional vertical confidence marker was displayed on top of the midpoint. Each dyad member made his private decision about which interval he thought contained the oddball target by moving the confidence marker to the left (first interval) or to the right (second interval) of the centre. The confidence marker could be moved along the line by up to five steps on either side, each step indicating higher confidence. There was no response time limit.

Next, the dyad member’s private responses (decision and confidence) were shared. In the case of agreement (i.e., if dyad members privately selected the same interval), they received feedback and continued to the next trial. But in the case of disagreement, one of the two dyad members was randomly prompted to make a joint decision on behalf of the dyad. In the non-verbal condition, the nominated dyad member made the joint decision only having access to the declared responses. In the verbal condition, the nominated dyad member also had the opportunity to verbally negotiate the joint decision with his partner. Dyad members were free to ignore each other’s confidence estimates at this stage of the experiment.

**Fig. 1:**
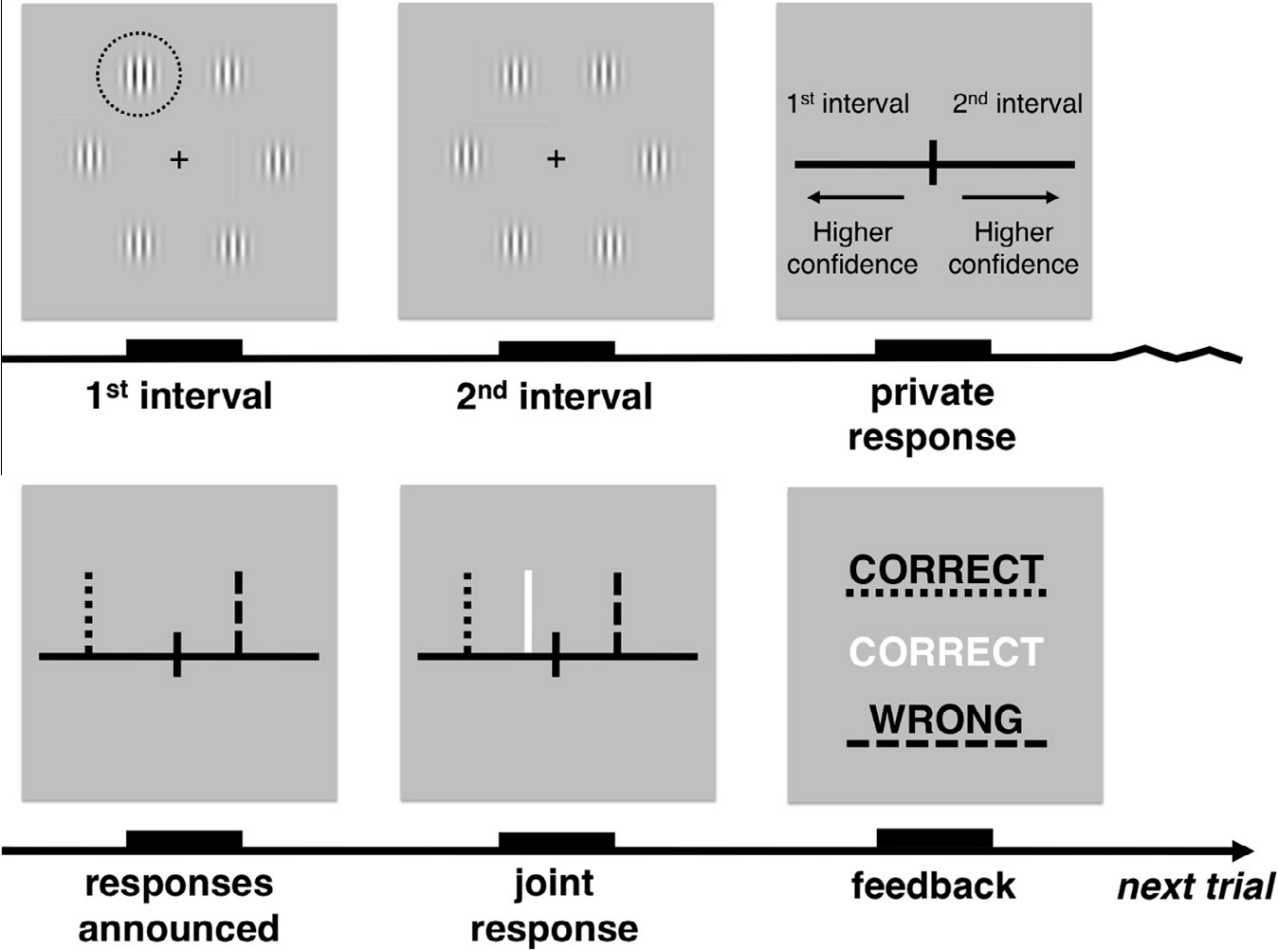
Schematic of one experimental trial. On each trial, dyad members briefly viewed two intervals, each containing six contrast gratings. In one of the two intervals, one of the six gratings had a slightly higher level of contrast (see encircled grating). After viewing the two intervals, dyad members privately used a vertical bar to indicate which interval they thought contained the target and their confidence in this decision. Their responses were then shared, with keyboard response shown in blue (dotted line) and mouse response shown in yellow (dashed line). If they independently selected the same interval, they received feedback (colour-coded) and continued to the next trial. If they privately selected different intervals, they were first asked to make a joint decision, using an additional white vertical bar.

After one practice block of 16 trials, two experimental sessions were conducted. Each session consisted of 8 blocks of 16 trials (128 trials in each session and 256 trials in total). Within each session, one dyad member responded with the keyboard (colour-coded as blue) and the other dyad member responded with the mouse (colour-coded as yellow). The dyad member using the keyboard controlled the confidence marker by pressing the ‘n’ (move left), the ‘m’ (move right), and the ‘b’ (submit response) buttons. The dyad member using the mouse controlled the confidence marker by pressing the ‘left’ (move left), the ‘right’ (move right), and the ‘scroll’ (submit response) buttons. Dyad members switched places (and hereby response device) at the end of the first session. See Fig. 1 for a schematic of an experimental trial.

## 3 Analysis

### 3.1 Computing MCS responses

We used Goodman & Kruskal’s gamma to test whether high (or low) confidence was associated with correct (or incorrect) decisions. As expected, the gamma coefficients (*mean* = .16, *SD* = .09) were significantly positive across dyad members, *t*(57) = 14.04, *p* < .001. For each dyad, we then derived the joint decisions advised by the MCS algorithm. In line with Koriat (2012), we first normalised the dyad members’ confidence estimates and then selected the decision of the more confident dyad member on each trial; the normalised confidence estimates, *c*_norm_, were related to the raw confidence estimates, *c*, via *c*_norm_ = (*c* − *μ*_c_dyad_)/*σ*_c_dyad_, where *μ_c_* and *σ_c_* were the mean and the standard deviation of the raw confidence estimates pooled from both dyad members.

### 3.2 Computing MRTS responses

Due to a programming error, the experimental code continued to sample the mouse response time until both mouse and keyboard responses had been registered. This error meant that mouse response times were either identical to keyboard response times (i.e., those trials in which the dyad member using the mouse actually made a faster response than the dyad member using the keyboard) or slower than keyboard response times (i.e., those trials in which the dyad member using the mouse made a slower response than the dyad member using the keyboard). Only including those trials in which dyad members responded with the keyboard (i.e., the first 128 trials for dyad member A and the last 128 trials for dyad member B – see Section 2.2), we regressed reaction times (milliseconds) against confidence to test whether faster decisions were associated with higher confidence. As expected, the unstandardised regression coefficients (*mean* *b* = −156.29) were significantly negative across dyad members, *t*(57) = −5.13, *p* < .001. Including all trials, for each dyad, we derived the joint decisions advised by the MRTS algorithm. As described above, we could infer which dyad member made the faster decision on each trial. The programming error meant that we could not test the effect of normalising reaction times.

### 3.3 Computing sensitivity

**Fig. 2:**
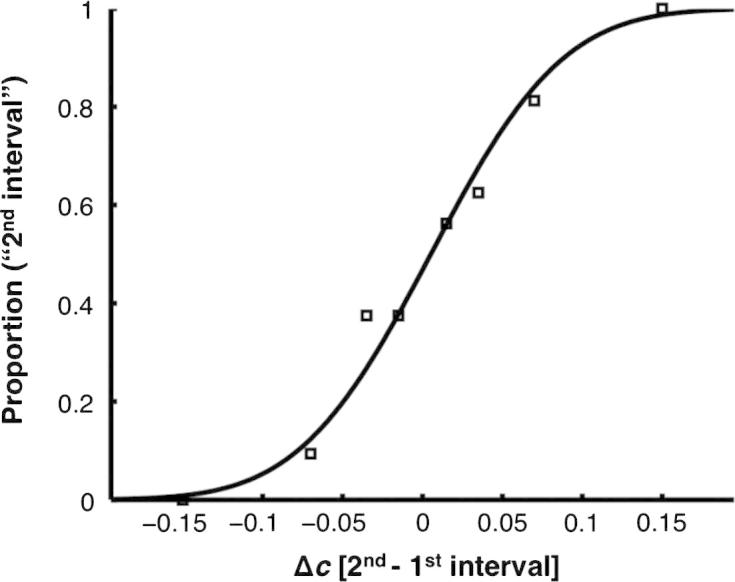
Example of a psychometric function. The *x-*axis shows Δ*c*, the contrast level in the second interval minus the contrast level in the first interval at the target location; negative values correspond to targets in the first interval and positive values correspond to targets in the second interval. The *y*-axis shows the proportion of trials in which the target was reported to be in the second interval. A highly sensitive observer would produce a steeply rising psychometric function with a large slope.

Psychometric functions were created for each dyad member and for the dyad (empirical or dummy) by plotting the proportion of trials in which the target was reported to be in the second interval against the contrast difference between the two intervals (i.e., the contrast level in the second interval minus the contrast level in the first interval at the target location). The steepness of the slope provides an estimate of sensitivity. More sensitive observers were, by definition, more ‘reliable’ in their estimates of contrast. See Fig. 2 for an example psychometric function.

The psychometric curves were fit with a cumulative Gaussian function whose parameters were bias, *b*, and variance, *σ*^2^. To estimate these parameters a probit regression model was employed using the *glmfit* function in MATLAB (Mathworks Inc.). A dyad member with bias *b* and variance *σ*^2^ would have a psychometric curve, denoted *P*(Δ*c*) where Δ*c* is the contrast difference between the two intervals, given by

$$P(\Delta c)=H\left(\frac{\Delta c+b}{\sigma }\right)\text{,}$$

where *H*(*z*) is the cumulative normal function

$$H(z)=\int_{-\infty }^{z}\frac{\mathrm{dt}}{{\left(2\pi \right)}^{1/2}}\exp\left[\frac{-t^{2}}{2}\right]$$

The psychometric curve, *P*(Δ*c*), corresponds to the probability of reporting that the second interval contained the target. Given the above definitions for *P*(Δ*c*), the variance is related to the maximum slope of the psychometric curve, denoted *S*, via

$$S=\frac{1}{{\left(2\pi \sigma^{2}\right)}^{1/2}}$$

A steep slope indicates small variance and thus highly sensitive performance. We used this measure to quantify individual and dyad (empirical or dummy) sensitivity.

We computed the similarity of dyad members’ reliabilities as the ratio of the sensitivity of the worse dyad member to that of the better dyad member (*S*_min_*/S*_max_), with values near zero corresponding to dyad members with very different reliabilities and values near one corresponding to dyad members of nearly equal reliability. We computed the collective outcome of the dyad (empirical or dummy) as the ratio of the sensitivity of the dyad (empirical or dummy) to that of the more sensitive dyad member (*S*_emp_*/S*_max_, *S*_MCS_*/S*_max_, and *S*_MRTS_*/S*_max_), with values below 1 indicating a collective loss and values above 1 indicating a collective benefit.

### 3.4 Computing metacognitive accuracy

**Fig. 3:**
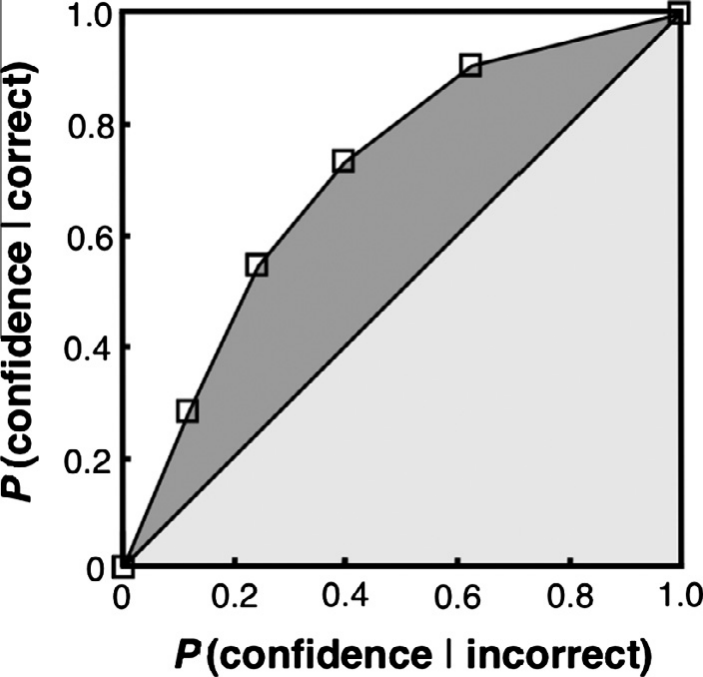
Example of an ROC curve. The *x-*axis and the *y*-axis show the cumulative probabilities *P*(confidence | incorrect) and *P*(confidence | correct), respectively. The sum of the shaded areas provides an estimate of metacognitive accuracy. The more bowed the curve, the higher the metacognitive accuracy (i.e. the probability of high confidence given correct rises more rapidly than the probability of high confidence given incorrect).

In line with previous studies (e.g., Fleming et al., 2010, and Song et al., 2011, who build on Galvin, Podd, Drga, & Whitmore, 2003; Kornbrot, 2006), we used the measure, *A_ROC_*, to quantify each dyad member’s metacognitive accuracy, and thereby the credibility of his confidence estimates. We first calculated the probabilities *p*(confidence = *k* | correct) and *p*(confidence = *k* | incorrect) for each level *k* of confidence. We then calculated the associated cumulative probabilities, *P*(confidence | correct) and *P*(confidence | incorrect), and plotted them against each other, thus yielding a Receiver Operating Characteristic (ROC) curve, with anchors at [0, 0] and [1, 1]. See Fig. 3 for an example ROC curve. Briefly, as the ROC curve shifts away from the major diagonal towards the upper left corner, the probability of high confidence given correct rises more rapidly than the probability of high confidence given incorrect. The area under the ROC curve (*A_ROC_*) thus provides an estimate of metacognitive accuracy. This area can be calculated geometrically as the sum of area between the ROC curve and the diagonal (dark grey) and the area of the half-square triangle below the diagonal (light grey):

$$A_{\mathrm{ROC}}=\overset{K-1}{\underset{i=1}{\sum }}\frac{\left(X_{i+1}-X_{i})(Y_{i+1}+y_{i}\right)}{2}+\frac{1}{2}\text{,}$$

where *X* and *Y* are the co-ordinates of the data points (squares in Fig. 3) and *K* is the number of confidence levels *k* (number of squares in Fig. 3).

### 3.5 Normalised versus raw confidence estimates

To test the effect of normalising confidence estimates before selecting the decision made with higher confidence, we also submitted raw confidence estimates to the MCS algorithm. The trials in which dyad members reported the same level of confidence but selected different intervals were resolved by randomly selecting the decision of one of the two dyad members; we note that there were no such confidence ties when submitting normalised confidence estimates to the MCS algorithm. To ensure that the random selection did not favour one of the dyad members by chance, for each dyad, we generated one hundred dummy dyads using raw confidence estimates and used their mean sensitivity (*S*_rawMCS_) to test the effect of normalising confidence estimates (*S*_MCS_/*S*_rawMCS_). See table 1 for a summary of the strategies for collective choice.

## 4 Results

As none of our interest measures showed significant differences between the non-verbal and the verbal conditions, we only report results based on data collapsed across both conditions. In addition, as none of these measures changed over time (i.e., from the first to the second session of the experiment), we only reported data collapsed across both experimental sessions.

### 4.1 The efficacy of the algorithms

**Fig. 4:**
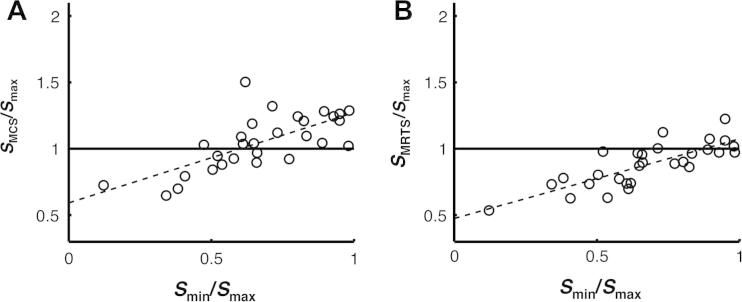
The collective benefit obtained from the MCS and MRTS algorithms depended on the similarity of dyad members’ sensitivities. The *x*-axis shows the ratio of the sensitivity of the worse dyad member relative to that of the better dyad member (*S*_min_/*S*_max_), with values near one corresponding to dyad members of nearly equal sensitivity. The *y*-axis shows (A) the ratio of the sensitivity of the MCS algorithm relative to that of the more sensitive dyad member (*S*_MCS_/*S*_max_) and (B) the ratio of the sensitivity of the MRTS algorithm relative to that of the more sensitive dyad member (*S*_MRTS_/*S*_max_), with values above one indicating a collective benefit over the more sensitive dyad member.

The similarity of dyad members’ sensitivities (*S*_min_*/S*_max_) significantly predicted the collective outcome of the MCS algorithm (*S*_MCS_*/S*_max_), *b* = .68, *t*(27) = 5.17, *p* < .001, and explained around 50% of the variance, *R*^2^ = 49.8, *F*_1,28_ = 26.74, *p* < .001 (see Fig. 4A). For similar dyad members, the MCS algorithm yielded a collective benefit (*S*_MCS_*/S*_max_ > 1 when *S*_min_*/S*_max_ > 0.6). However, for dissimilar dyad members, the MCS algorithm yielded a collective loss (*S*_MCS_*/S*_max_ < 1 when *S*_min_*/S*_max_ < 0.6). The similarity of dyad members’ sensitivities also significantly predicted the collective outcome of the MRTS algorithm (*S*_MRTS_*/S*_max_), *b* = .59, *t*(27) = 4.69, *p* < .001, and explained around 45% of the variance, *R*^2^ = 44.9, *F*_1,28_ = 22.02, *p* < .001 (see Fig. 4B). However, the MRTS only yielded a collective benefit for five dyads. The MCS algorithm was superior to the MRTS algorithm, both when normalised confidence estimates, *t*(28) = 4.99, *p* < .001, and raw confidence estimates, *t*(28) = 6.26, *p* < .001, were used as its input.

### 4.2 The relative benefit of interaction over the algorithms

**Fig. 5:**
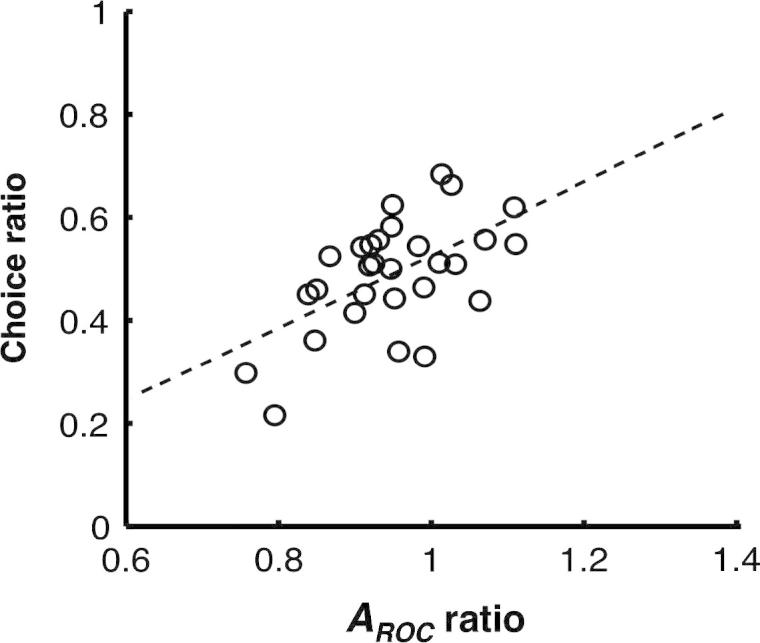
Interacting dyad members took each other’s metacognitive ability into account when making joint decisions. The *x*-axis shows the ratio of the *A_ROC_* of dyad member A relative to that of dyad member B. The *y*-axis shows the fraction of disagreement trials in which the dyad followed the decision of dyad member A instead of that of dyad member B.

To test whether interacting dyad members used the credibility of each other’s confidence estimates to guide their joint decisions, we regressed the fraction of disagreement trials in which the dyad eventually followed the decision of dyad member A instead of that of dyad member B (the choice ratio) against the ratio of the *A_ROC_* for dyad member A relative to that of dyad member B (the *A_ROC_* ratio). The *A_ROC_* ratio significantly predicted the choice ratio, *b* = .70, *t*(27) = 3.58, *p* < .001, and explained around 30% of the variance, *R*^2^ = .32, *F*_1,28_ = 12.78, *p* < .001 (see Fig. 5).

**Fig. 6:**
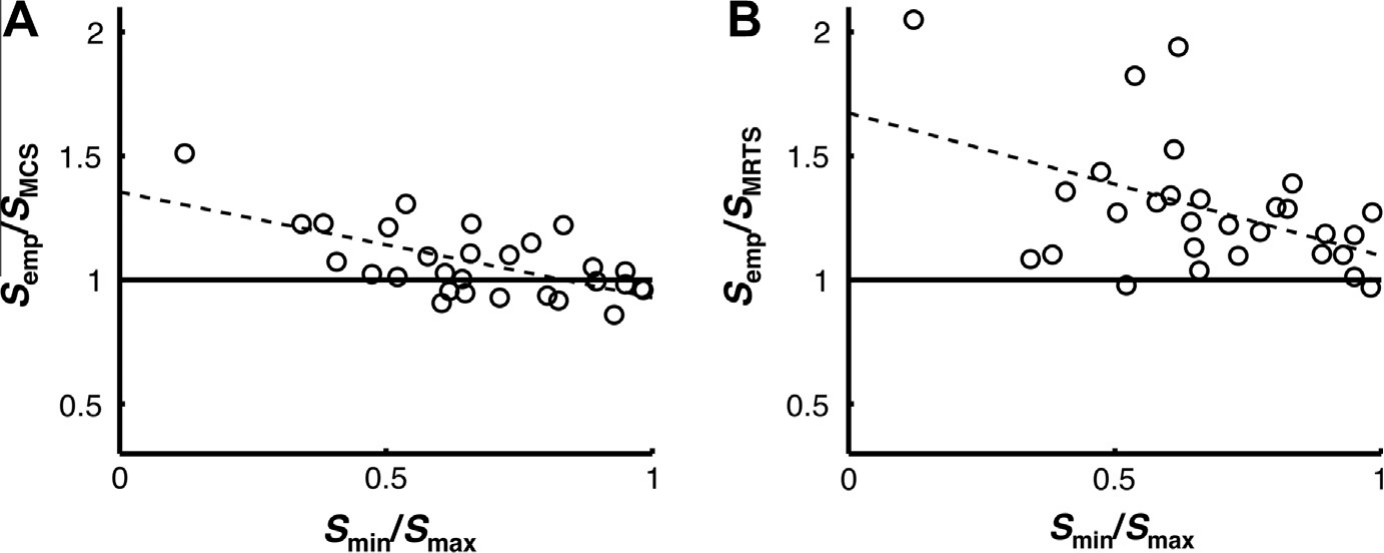
The relative benefit for interaction over the MCS and MRTS algorithms depended on the similarity of dyad members’ sensitivities. The *x*-axis shows the ratio of the sensitivity of the worse dyad member relative to that of the better dyad member (*S*_min_/*S*_max_), with values near one corresponding to dyad members of nearly equal sensitivity. The *y*-axis shows (A) the ratio of the sensitivity of the empirical dyad relative to that of the MCS algorithm (*S*_emp_/*S*_MCS_) and (B) the ratio of the sensitivity of the empirical dyad relative to that of the MRTS algorithm (*S*_emp_/*S*_MRTS_), with values above one indicating a relative benefit for interaction.

The similarity of dyad members’ sensitivities significantly predicted the performance of the empirical dyads relative to that of the MCS algorithm (*S*_emp_*/S*_MCS_), *b* = −.43, *t*(27) = −4.21, *p* < .001, and explained around 40% of the variance, *R*^2^ = .40, *F*_1,28_ = 17.73, *p* < .001 (see Fig. 6A). For similar dyad members, there was no relative benefit for interaction over the MCS algorithm (*S*_emp_*/S*_MCS_ ≈ 1 when *S*_min_*/S*_max_ > 0.8). However, for dissimilar dyad members, there was a relative benefit for interaction over the MCS algorithm (*S*_emp_*/S*_MCS_ *>* 1 when *S*_min_*/S*_max_ < 0.8). The similarity of dyad members’ sensitivities also significantly predicted the performance of the empirical dyads relative to that of the MRTS algorithm (*S*_emp_*/S*_MCS_), *b* = −.651, *t*(27) = −2.20 *p* = .037, and explained around 15% of the variance, *R*^2^ = .15, *F*_1,28_ = 4.83, *p* = .037 (see Fig. 6B). However, the MRTS algorithm only (marginally) outperformed two of the empirical dyads.

### 4.3 The effect of normalising confidence estimates

**Fig. 7:**
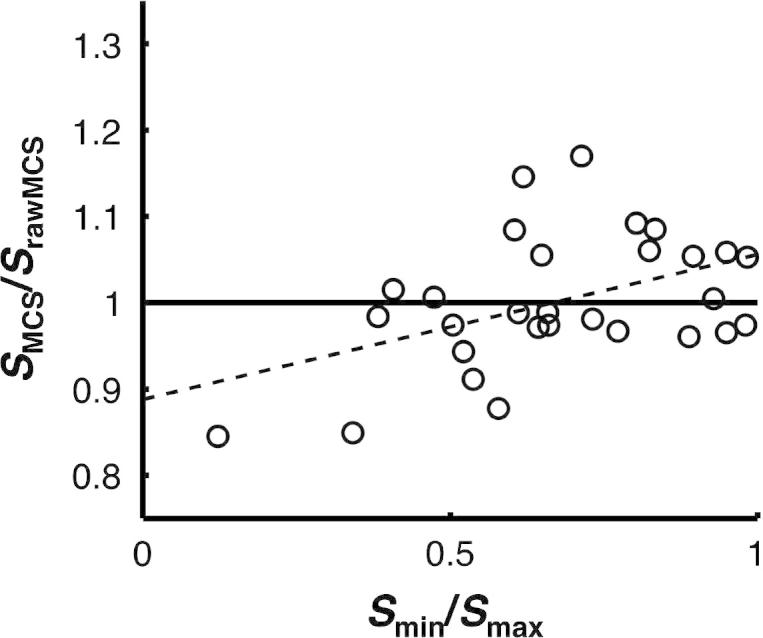
The effect of normalising confidence estimates depended on the similarity of dyad members’ sensitivities. The *x*-axis shows the ratio of dyad members’ sensitivities (*S*_min_/*S*_max_), with values near one corresponding to dyad members of nearly equal sensitivity. The *y*-axis shows the ratio of the sensitivity of the MCS algorithm using normalised confidence estimates relative to that of the MCS algorithm using raw confidence estimates (*S*_MCS_/*S*_rawMCS_), with values above the horizontal line indicating a relative benefit for normalising confidence estimates.

The similarity of dyad members’ sensitivities significantly predicted the effect of normalising confidence estimates (*S*_MCS_/*S*_rawMCS_), *b* = .17, *t*(27) = 2.70, *p* = .012, and explained around 20% of the variance, *R*^2^ = .21, *F*_1,28_ = 7.31, *p* = .012 (see Fig. 7). For similar dyad members, there was a relative benefit for normalising confidence estimates (*S*_MCS_/*S*_rawMCS_ *>* 1 when *S*_min_*/S*_max_ > 0.6). However, for dissimilar dyad members, there was a relative cost to normalising confidence estimates (*S*_MCS_/*S*_rawMCS_ < 1 when *S*_min_*/S*_max_ < 0.6). We note that this effect was relatively subtle (cf. change of scale on *y*-axis in Fig. 7), and that the relative benefit of interaction over the MCS algorithm was not affected using raw, instead of normalised confidence estimates, as input to the MCS algorithm.

## 5 Discussion

Sharing of confidence as a strategy for combining individual opinions into group decisions has been established in a wide range of contexts. This tendency to evaluate the reliability of information by the confidence with which it is expressed has been termed the ‘confidence heuristic’. In this study, we tested two simple ways of implementing the confidence heuristic in the context of a collective perceptual decision-making task: the MCS algorithm, which opts for the decision made with higher confidence, and the MRTS algorithm, which opts for the faster decision, exploiting a negative correlation between confidence and reaction time. Our findings have important implications for the use of heuristics for collective choice and for models of confidence and collective decision-making.

### 5.1 The efficacy of the MCS and the MRTS algorithms

According to signal detection theory (Macmillan & Creelman, 2005), an observer’s perception of a visual event can be thought of as a random sample from some distribution. In our case, the mean (*μ*) of the distribution – which we take to be Gaussian – is given by the actual contrast difference between the two intervals at the target location (Δ*c* – see Fig. 2) and the standard deviation (*σ*) of the distribution specifies the level of noise in the observer’s perceptual system. The sign of the random sample, which we denote, $\overset{∼}{\Delta c}$, specifies whether the observer perceived the target in the first interval ($\overset{∼}{\Delta c}<0$) or in the second interval ($\overset{∼}{\Delta c}>0$). As such, a reliable decision is characterised by a large $\overset{∼}{\Delta c}$ and a small *σ*.

Assuming that dyad members can estimate the level of noise in their perceptual system, the Weighted Confidence Sharing (WCS) model (Bahrami et al., 2010) proposes that communicated confidence is a monotonic function of the $\overset{∼}{\Delta c}/\sigma$ ratios (i.e., a *z*-score) associated with their perceptions, with the same monotonic function for both dyad members. Crucially, the $\overset{∼}{\Delta c}/\sigma$ ratios relates directly to the probability that the respective decisions are correct. The optimal strategy for collective choice is therefore to follow the dyad member with the $\overset{∼}{\Delta c}/\sigma$ ratio of larger magnitude. Under this decision strategy, collective performance is a linear function of the similarity of dyad members’ sensitivities (i.e., the reliability of their decisions), with low similarity leading to a collective loss (see Supplementary materials to Bahrami et al., 2010, for mathematical details).

The MCS algorithm effectively implements the WCS model by selecting the decision made with higher confidence (cf. the $\overset{∼}{\Delta c}/\sigma$ ratio of higher magnitude) on each trial. We therefore predicted that the MCS algorithm would be linearly dependent on the similarity of dyad members’ reliabilities. To the extent that confidence was negatively correlated with reaction time – a typical finding when responses are not speeded (e.g., Patel et al., 2012; Pleskac & Busemeyer, 2010; Vickers & Packer, 1982) – we also predicted that the MRTS algorithm would be linearly dependent on the similarity of dyad members’ reliabilities.

As expected, the MCS and the MRTS algorithms only yielded 2HBT1 effects for dyad members of nearly equal reliability. However, despite a negative correlation between confidence and reaction time, the MCS algorithm markedly outperformed the MRTS algorithm. The superiority of the MCS algorithm to the MRTS algorithm could be due to differences in the ‘pre-processing’ of their input. While we could submit both normalised and raw confidence estimates to the MCS algorithm, we could only submit raw reaction times to the MRTS algorithm because of a programming error (see Section 3.2). Since individuals vary with respect to their average response speed, pooling raw reaction times from two individuals might corrupt the link between confidence and reaction time. However, the MCS algorithm outperformed the MRTS algorithm even when raw confidence estimates were used as its input, suggesting that reaction time may be a very noisy substitute for confidence. The sign of the correlation between confidence and reaction time has been found to depend on response demands, with a *negative* correlation in the absence of speed pressure and a *positive* correlation under speed pressure (see Pleskac & Busemeyer, 2010, for a computational account of this phenomenon). While no speed pressure was enforced in the current task, dyad members may have paced their responses on a subset of trials, thus corrupting the negative correlation between confidence and reaction time.

We note that, outside the context of the MCS and the MRTS algorithms, the normalisation of confidence estimates has a more straightforward interpretation than the normalisation of reaction times. The normalisation of confidence estimates is intended to remove biases in the use of a scale – here, how an internal variable is mapped onto a confidence scale – and could potentially capture important aspects of collective decision-making (see Section 5.3). It is less clear how the normalisation of reaction times would translate into other contexts. Taken together, our findings show that heuristics for collective choice are susceptible to individual differences in reliability, and suggests that reaction time cannot be substituted for confidence without incurring a considerable collective accuracy cost. In this light, we will limit the remainder of the Discussion to the MCS algorithm.

### 5.2 The relative benefit of interaction over the MCS algorithm

If the assumptions of the WCS model were satisfied, the responses advised by the MCS algorithm should be just as accurate as those reached by dyad members through interaction. However, the ability to estimate the reliability of one’s own decisions (cf. the level of noise in one’s perceptual system) shows substantial individual differences (e.g., Fleming et al., 2010; Song et al., 2011); this ability is typically referred to as metacognitive ability and quantified as metacognitive accuracy. While the MCS algorithm is prone to error when people misestimate the reliability of their own decisions, interacting individuals may take such misestimates into account. For example, one study has shown that mock jurors find witnesses who are confident about erroneous testimony less credible than witnesses who are not confident about it (Tenney et al., 2007). We predicted that interacting dyad members would take into account the credibility of each other’s confidence estimates when making their joint decisions, and that interaction would be relatively more beneficial than the algorithms for dissimilar dyad members, because they have more to lose from following the more confident but less competent of the two.

As for the first prediction, the fraction of disagreement trials in which the dyad followed dyad member A instead of dyad member B depended on their relative metacognitive accuracy (here measured as *A_ROC_* – see Section 3.4), indicating that dyad members used the credibility of each other’s confidence estimates to guide their joint decisions (see Fig. 5). As for the second prediction, interaction was more robust than the MCS algorithm to differences in reliability. For similar dyad members, the decisions reached through interaction were no more accurate than those advised by the MCS algorithm. However, for dissimilar dyad members, the decisions reached through interaction were considerably more accurate than those advised by the MCS algorithm; this was true irrespective of whether normalised or raw confidence estimates were submitted to the MCS algorithm. While models of collective decision-making have identified the ‘arbitration’ of confidence estimates as key to collective performance (e.g., Bahrami et al., 2010; Koriat, 2012), our findings suggests that the ‘weighting’ of confidence estimates is equally important for collective performance. Without taking the credibility of confidence estimates into account, the MCS algorithm cannot fully replace interaction in collective decision-making.

Our study highlights the social heterogeneity of credibility as an interesting avenue for computational research: how do we estimate the credibility of each other’s opinions, and how good are we at doing so? We note that the relative benefit of interaction over the MCS algorithm need not necessarily result from dyad members discounting the opinion of the dyad member with lower metacognitive accuracy. More specifically, while dyad members may assign less weight to the opinion of the more confident but less competent member (“bad but doesn’t know it”), they may also assign more weight to the opinion of the less confident but more competent dyad member (“good but does know it”). We believe that computational models of social learning (e.g., Behrens, Hunt, Woolrich, & Rushworth, 2008) are needed to tease apart such decision strategies.

### 5.3 The effect of normalising confidence estimates

Even if dyad members have access to the $\overset{∼}{\Delta c}/\sigma$ ratios associated with their perceptions, they still have to solve the problem of how to map specific $\overset{∼}{\Delta c}/\sigma$ ratios onto specific levels of confidence. This ‘mapping problem’ may explain why the relative benefit of normalising confidence estimates depended on the similarity of dyad members’ reliabilities. If dyad members mapped *similar* $\overset{∼}{\Delta c}/\sigma$ ratios (i.e., $\overset{∼}{\Delta c}/\sigma$ ratios of similar magnitudes) onto *different* levels of confidence, then normalising confidence estimates would re-map those ratios onto *similar* levels of confidence, thus improving the performance of the MCS algorithm. However, if dyad members mapped *different* $\overset{∼}{\Delta c}/\sigma$ ratios (i.e., $\overset{∼}{\Delta c}/\sigma$ ratios of different magnitudes) onto *similar* levels of confidence, then normalising confidence estimates would strengthen this ‘erroneous’ mapping, thus decreasing the performance of the MCS algorithm. In this perspective, similar dyad members (who had $\overset{∼}{\Delta c}/\sigma$ ratios of similar magnitudes) did not map their $\overset{∼}{\Delta c}/\sigma$ ratios onto sufficiently *similar* confidence distributions, whereas dissimilar dyad members (who had $\overset{∼}{\Delta c}/\sigma$ ratios of different magnitudes) did not map their $\overset{∼}{\Delta c}/\sigma$ ratios onto sufficiently *different* confidence distributions. We believe that computational models of decision confidence (e.g., Kepecs, Uchida, Zariwala, & Mainen, 2008) are needed to address how individuals solve – or should solve – the mapping problem in social contexts.

A linguistic analysis (Fusaroli et al., 2012) of the conversations in Bahrami et al.’s (2010) study showed that dyad members who aligned their linguistic confidence estimates accrued larger 2HBT1 effects. More specifically, the dyad members who used confidence expressions from the same linguistic set (e.g., both using gradients of “sure”, such as “very sure” and “slightly sure”) performed better than those who used confidence expressions from different linguistic sets (e.g., gradients of “sure” versus “know”). If linguistic alignment involves normalisation in the statistical sense described here, then the positive effect of linguistic alignment could depend on the similarity of dyad members’ reliabilities. However, linguistic interaction may offer other ways of solving the mapping problem. For example, individuals may carve a fine-grained scale of confidence that fits the continuous nature of their $\overset{∼}{\Delta c}/\sigma$ ratios (e.g., the linguistic sets in Fusaroli et al., 2012, contained on average 18 items). Moreover, individuals may discuss each other’s use of the confidence scale (e.g., what it means to be “very sure”), thus preventing ‘erroneous’ mapping of their $\overset{∼}{\Delta c}/\sigma$ ratios. Lastly, individuals have more experience with mapping internal variables onto linguistic expressions than with mapping internal variables onto numerical estimates. Indeed, probing people’s perceptual experiences with linguistic expressions seems to give a more accurate measure of their metacognitive ability (Overgaard & Sandberg, 2012).

### 5.4 Will the findings generalise beyond the current study?

#### 5.4.1 Role of feedback

All participants received feedback about the accuracy of each decision, and could thus directly evaluate the credibility of each other’s confidence estimates. Indeed, previous research has shown that diagnostic feedback helps groups of individuals to identify their more accurate members (Henry, Strickland, Yorges, & Ladd, 1996). The relative benefit of interaction over the MCS algorithm might therefore not persist in the absence of diagnostic feedback. However, using the same visual perceptual task, Bahrami et al. (2012b) found that diagnostic feedback was not necessary for the accumulation of a 2HBT1 effect – diagnostic feedback only appeared to accelerate the process – indicating that individuals may rely on other signals when they learn the credibility of each other’s confidence estimates. The identification and incorporation of these signals will pose a major challenge for dynamic models of collective decision-making.

#### 5.4.2 Role of familiarity

Here, for each dyad, one participant was recruited, and then asked to bring along a friend to the study. Dyad members might thus have used their interpersonal history to establish the credibility of each other’s confidence estimates. However, using a similar task, but in the domain of approximate numeration, Bahrami, Didino, Frith, Butterworth, and Rees (2013) found that familiarity had little impact on collective performance, suggesting the dyad members evaluated the credibility of each other’s confidence estimates in the context of their current task performance. While there is no evidence for a main effect of familiarity on collective performance, it may be the case that familiarity matters more for dissimilar than similar dyad members.

#### 5.4.3 Non-perceptual domains

Research has shown that individuals are ‘overconfident’ about the accuracy of their knowledge-based judgements but ‘underconfident’ about the accuracy of their perceptual judgements (see Harvey, 1997, for a review). These discrepant patterns of confidence have led to the hypothesis that different types of information determine confidence in knowledge and perception. For example, Juslin and Olsson (1997) propose a model of confidence in which perceptual judgements are dominated by ‘Thurstonian’ uncertainty, internal noise such as stochastic variance in the sensory systems, whereas knowledge-based judgments are dominated by ‘Brunswikian’ uncertainty, external noise such as less-than-perfect correlations between features in the environment. This dissociation raises issues as to whether confidence can be used as a proxy for the reliability of decisions in non-perceptual domains. However, direct comparison of knowledge-based and perceptual judgements has found evidence for a common basis of confidence (e.g., Baranski & Petrusic, 1994; Pallier et al., 2002), suggesting that the efficacy of the MCS algorithm will generalise to non-perceptual domains (but see Koriat, 2012, for exceptional environments).

## 6 Conclusion

Using a visual perceptual task, we tested whether a confidence heuristic could replace interaction in collective decision-making – either directly, by opting for the judgement made with higher confidence, or indirectly, by opting for the faster judgement. We found that reaction time could not be substituted for confidence without incurring a considerable collective accuracy cost. Moreover, we found that, for individuals of nearly equal reliability, the decisions advised by the confidence heuristic were just as accurate as those reached through interaction, but for individuals with different reliabilities, the decisions advised by the confidence heuristic were less accurate than those reached through interaction. Relatedly, we found that interacting individuals took into account the credibility of each other’s confidence when making their joint decisions, presumably making them less susceptible to those situations in which the more confident was the less competent group member. Lastly, we found that normalising confidence estimates increased the efficacy of the confidence heuristic for individuals of nearly equal reliability but had the opposite effect for individuals with different reliabilities. Taken together, these findings highlight two issues for future research: First, how do individuals estimate the credibility of each other’s opinions, and how good are they at doing so? Second, how do individuals map ‘internal’ variables onto ‘external’ variables (e.g., map the $\overset{∼}{\Delta c}/\sigma$ ratios of their sensory representations onto shareable confidence estimates) and how should they solve this mapping problem so as to facilitate collective performance?

## Funding

This work was supported by the Calleva Research Centre for Evolution and Human Sciences (DB, JYFL), the European Research Council Starting Grant NeuroCoDec 309865 (BB), the Danish Council for Independent Research – Humanities (KT, RF), the EU-ESF program Digging the Roots of Understanding DRUST(KT, RF), the European UnionMindBridge Project (DB, KO, AR, CDF, BB), the Gatsby Charitable Foundation (PEL), and the Wellcome Trust (GR).

## Figures and Tables

**Table 1:** Strategies for collective choice.

Strategy	Abbreviation	Explanation
Normalised Maximum Confidence Slating	MCS	The joint decision was based on the dyad member with the higher normalised confidence estimate
Raw Maximum Confidence Slating	rawMCS	The joint decision was based on the dyad member with the higher raw confidence estimate
Minimum Reaction Time Slating	MRTS	The joint decision was based on the dyad member with the shorter reaction time
Empirical dyad	emp	A randomly chosen dyad member made the joint decision after having access to the other dyad member’s raw confidence estimate (non-verbal condition) or also having the opportunity to discuss with the other dyad member (verbal condition)
